# Synthetic Genistein Glycosides Inhibiting EGFR Phosphorylation Enhance the Effect of Radiation in HCT 116 Colon Cancer Cells

**DOI:** 10.3390/molecules191118558

**Published:** 2014-11-13

**Authors:** Aleksandra Gruca, Zdzisław Krawczyk, Wiesław Szeja, Grzegorz Grynkiewicz, Aleksandra Rusin

**Affiliations:** 1 Department of Organic Chemistry, Bioorganic Chemistry and Biotechnology, Silesian University of Technology, Krzywoustego 4, 44-100 Gliwice, Poland; E-Mails: gruca.aleksandra@gmail.com (A.G.); wieslaw.szeja@adres.pl (W.S.); 2 Center for Translational Research and Molecular Biology of Cancer, Maria Sklodowska-Curie Memorial Cancer Center and Institute of Oncology, Gliwice Branch, Wybrzeze Armii Krajowej 15, 44-100 Gliwice, Poland; E-Mail: krawczyk@io.gliwice.pl; 3 Pharmaceutical Research Institute, Rydygiera 8, 01-793 Warsaw, Poland; E-Mail: g.grynkiewicz@ifarm.eu

**Keywords:** genistein glycoconjugates, epidermal growth factor receptor inhibition, radiotherapy, radiosensitization

## Abstract

The need to find new EGFR inhibitors for use in combination with radiotherapy in the treatment of solid tumors has drawn our attention to compounds derived from genistein, a natural isoflavonoid. The antiproliferative potential of synthetic genistein derivatives used alone or in combination with ionizing radiation was evaluated in cancer cell lines using clonogenic assay. EGFR phosphorylation was assessed with western blotting. Genistein derivatives inhibited clonogenic growth of HCT 116 cancer cells additively or synergistically when used in combination with ionizing radiation, and decreased EGFR activation. Our preclinical evaluation of genistein-derived EGFR inhibitors suggests that these compounds are much more potent sensitizers of cells to radiation than the parent isoflavonoid, genistein and indicate that these compounds may be useful in the treatment of colon cancer with radiation therapy.

## 1. Introduction

The epidermal growth factor receptor (EGFR) plays an important role in response of cancer cells to therapy. The binding of a ligand induces its homo- or heterodimerization with other members of the ErB family and leads to trans-autophosphorylation of tyrosines in the intracellular domain. This initiates phosphorylation of other intracellular proteins and adaptors, triggering activation of multiple signaling cascades governing cell proliferation, survival, invasion, adhesion, cell cycle progression, cell motility and DNA repair [1,2]. In cancer cells, EGFR signaling is often enhanced due to overexpression of ligands, the elevated level of the receptor, and the presence of activating mutations. The association between over-activation of EGFR and a worsened prognosis [3,4,5] has motivated many researchers to develop clinically useful inhibitors of its tyrosine kinase activity (TKI). Although the results of several clinical trials with use of available TKIs alone or in combination with chemo- and radiotherapy are encouraging [6,7,8], there are examples of disappointing outcomes [9]. Therefore, elaboration of new TKIs/new therapeutic modalities using known TKIs are strongly deserved.

The alternative to EGFR inhibitors currently used in cancer therapy, particularly with intended use in combination with other therapeutic modalities, may be non-toxic products of natural origin, such as general tyrosine kinase inhibitor, genistein or its derivatives. One of early observations relevant to possible anticancer applications of genistein was inhibitory activity of this compound on tyrosine kinases, including c-src i v-abl and EGFR [10]. Recent analysis suggested that genistein inhibited the activity of tyrosine kinase EGFR, PDGFR, insulin receptor, Abl, Fgr, Itk, Fyn and Src [11].

Genistein was shown to potentiate the effects of other tyrosine kinase inhibitors such as erlotinib, gefitinib and the monoclonal antibody cetuximab [12,13,14]. This isoflavonoid was reported to enhance the effects of radiotherapy in different cellular and animal models [15,16,17,18]. There were also indications that genistein played a protecting role against side effects evoked by radiotherapy, such as lung injury or cardiac dysfunction [19]. Genistein partially reduced the extent of fibrosis developing in mouse lungs after irradiation with no evidence of protection of small tumors in the lung [19]. Using genistein along with irradiation prevented the incidence of delayed lung injuries in mice [20] and aided early recovery of hematopoietic cells by stimulation of cytokins production [21].

Genistein inhibits the activity of tyrosine kinases at concentrations above 10 µM, which is difficult to achieve *in vivo*. Some efforts were made to obtain derivatives of genistein showing enhanced anti-kinase activity, but so far they have largely been unsuccessful [22,23]. The relatively simple structure of the genistein molecule and the many possibilities of its derivatization offer ample space for obtaining derivatives with improved activity or affecting new molecular targets [24]. In the previous work, we described glycoconjugates of genistein inhibiting cancer cell proliferation more efficiently than genistein [25,26,27,28,29]. The aim of this work was to evaluate EGFR tyrosine kinase inhibition by selected genistein derivatives and to explore the biological effects of these compounds used in combination with ionizing radiation.

## 2. Results and Discussion

### 2.1. Inhibition of Clonogenic Cell Survival

**Figure 1:**
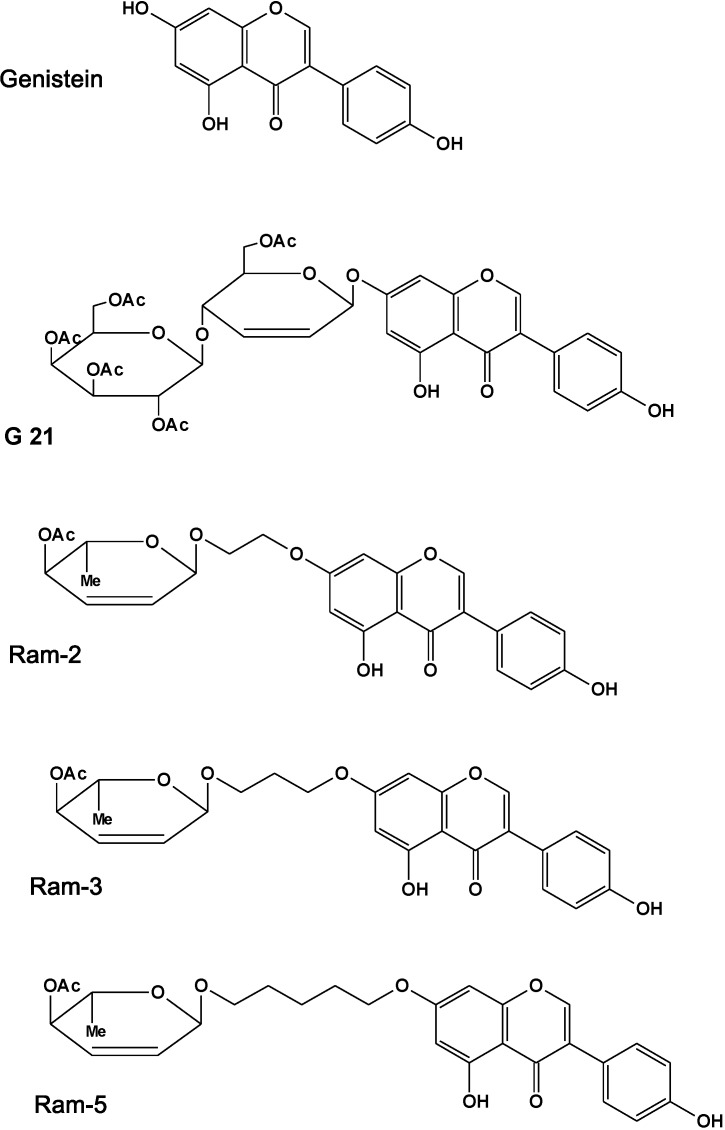
Chemical structure of genistein, G21, Ram-2, Ram-3, and Ram-5.

This work extends our previous findings of antiproliferative activity of sugar derivatives of genistein in cancer cell lines. Although the role a sugar moiety in many drugs seems to be important for their biological activity, only few selected categories of natural glycosides (e.g., different classes of antibiotics) have been examined in details for their pharmacological activity, and the studies comparing side by side activities of glycosides and their aglycons are scarce. In order to determine whether genistein and its four sugar derivatives (Figure 1) can potentiate the cytotoxic effect of radiation in cancer cells exhibiting high expression of EGFR protein and EGFR-dependent autocrine proliferation [30] we performed the clonogenic assay in the HCT 116 human colorectal line.

**Figure 2:**
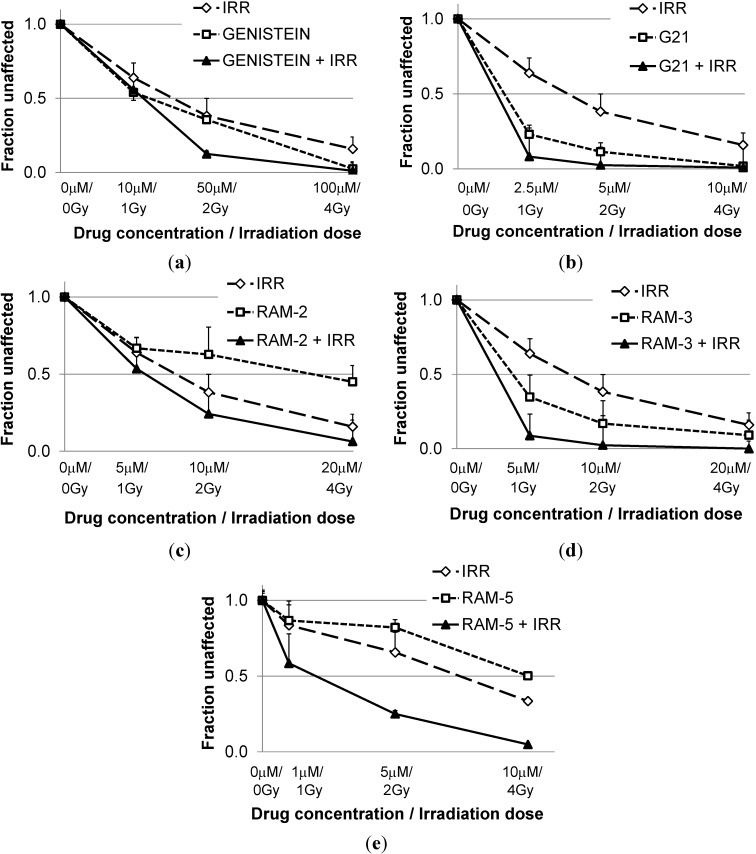
Clonogenic survival of HCT 116 cell line after treatment with growing concentrations of genistein (**a**) or its derivatives, G21 (**b**), Ram-2 (**c**), Ram-3(**d**) and Ram-5 (**e**) and growing dose of radiation.

Concentration and dose-dependent inhibition of clonogenic survival of HCT 116 was assessed in a series of experiments, in which cells pretreated for 24 h with genistein or its derivatives were irradiated with a linear accelerator (Figure 2). To determine the type of additive effects from the tested derivatives and irradiation, the combination index (CI) was calculated (Table 1). This revealed that values of combination indices tended to decrease with growing effect. Genistein derivatives were much better sensitizers of cells to radiation then genistein. At the effect levels expressed as ED_50_ and ED_75_, interactions of genistein derivatives and radiation were mostly additive (while genistein was antagonistic), and at ED_90_ they turned out to be synergistic (CI < 0.7) (whereas genistein with radiation acted only additively). Here, we confirmed our previous results showing that modification of genistein with certain hexenoses may enhance antiproliferative activity of a derivative.

**Table 1:** Values of combination index (CI) for drug-irradiation interactions at different effects: ED_50_, ED_75_ and ED_90_.

	CI * Values Calculated for Different Effects		
	ED_50_	ED_75_	ED_90_
Genist/Irr	1.75 ± 0.25	1.36 ± 0.12	1.10 ± 0.19
G21/Irr	1.01 ± 0.54	0.86 ± 0.44	0.77 ± 0.35
Ram-2/Irr	1.14 ± 0.05	0.7±0.01	0.54±0.05
Ram-3/Irr	1.22 ± 0.16	0.72 ± 0.15	0.43 ± 0.12
Ram-5/Irr	0.63 ± 0.11	0.35 ± 0.08	0.21 ± 0.09

* CI is a quantitative measure of the degree of interaction between different treatments. When CI is equal to 0.9–1.1 it denotes additivity; If CI is greater than 1.1—antagonism; CI values between 0.9 and 0.7 indicate slight synergism; CI values less than 0.7—synergism. CI values were calculated according to the Chou and Talalay mathematical model for drug interactions [27] using the Calcusyn software on the base of the results of clonogenic assays. Data represent mean CI calculated from three independent experiments ± standard deviations.

Previous reports have indicated that genistein functions as a radiosensitizer in different cancer cells [15,16,17,18,31,32]. In this work we found that genistein used in combination with ionizing radiation produced weakly antagonistic or additive antiproliferative effects in HCT 116 cells, whereas its synthetic, glycosidic derivatives were much better radiosensitizers and acted synergistically when combined with radiation. The derivatives Ram-3 and Ram-5 were particularly efficient in cessation of clonogenic potential of irradiated cells. The most often cited biological pathways affected by genistein that lead to potentiation of radiotherapeutic effects are: inhibition of Nfkb, G2/M block of the cell cycle, inhibition of AKT and inhibition of tyrosine kinases [11,18]. However, the well documented activity of genistein on other molecular targets, such as estrogen receptors, DNA topoisomerases, protein kinase C or FGFR-1 may also influence radiotherapeutic response [33,34]. Although modulating effects of the tested derivatives on the pathways mentioned above are very likely, the presented work was focused on inhibition of EGFR by genistein derivatives, and evaluation of their preclinical activity in combination with ionizing radiation.

It is worth mentioning that the tested derivatives were apparently non-toxic for confluent and differentiated Caco-2 monolayers (*in vitro* model of human intestine) and they inhibited proliferation of normal cells (human keratinocyte cell line HaCaT) at higher concentration when compared with cancer cells.

### 2.2. Inhibition of EGFR Phosphorylation in Cancer Cells Treated with Genistein Derivatives

**Figure 3:**
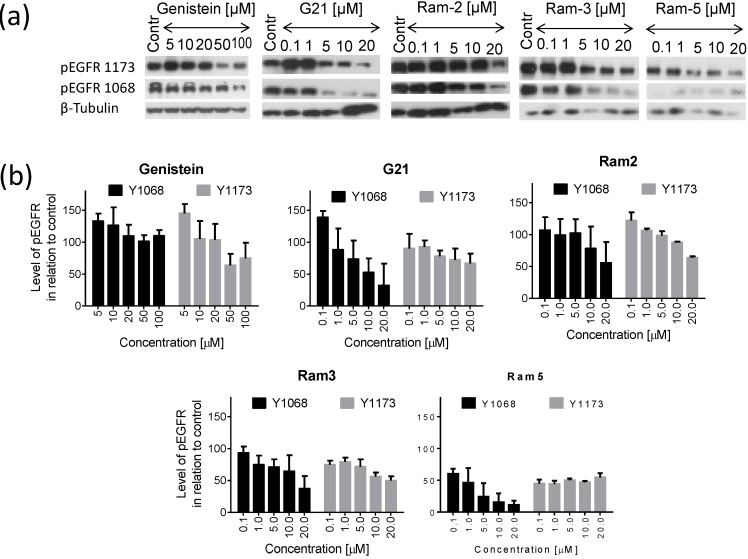
Phosphorylation of EGFR in HCT 116 cells treated with genistein or its derivatives for 24 h. (**a**) western blots showing pEGFR 1068, pEGFR 1173 level and β-tubulin (used as a loading control); (**b**) charts presenting the results of densitometry. Bars show the mean values ± standard deviations of the band density normalized to the loading control in relation to untreated control (Contr) normalized to the loading control. Data from at least three independent experiments.

We analyzed the relative amount of pEGFR (Y1068 and Y1173) in cells treated for 24 h (Figure 3). Ram-2, Ram-3 and Ram-5 inhibited phosphorylation of EGFR both at the tyrosine 1068 and 1173 in a dose dependent manner, albeit G21 and genistein showed a biphasic response and at low concentrations the level of EGFR phosphorylation was higher than in the untreated control. The level of EGFR phosphorylation was reduced remarkably by Ram-5, which showed its inhibitory activity at very low concentration (0.1 µM). The general conclusion from the presented data is that all the tested sugar derivatives of genistein reduced the level of EGFR phosphorylation after 24 h treatment more efficiently than a parent drug, genistein. We also performed these experiments in DU 145 cell line and found a similar pattern of inhibition of EGFR phosphorylation, proving that the effect of the tested compounds is not limited to one cell line.

Inhibition of EGFR and several other tyrosine kinases by genistein was discovered a long time ago [28]. We currently know that genistein inhibits the activity of EGFR, PDGFR, insulin receptor, Abl, Fgr Itk, Fyn and Src [11]. Inhibition of EGFR by this isoflavonoid was confirmed in other studies [35]. Whereas a systematic search for optimum correlation between structure-activity relationship was performed for flavonoids as inhibitors of p56^lck^ kinase [36,37], no such comprehensive study was done for tyrosine kinases and isoflavonoids. Experimental data obtained shortly after finding the inhibitory activity of genistein against EGFR indicate some structural features, relevant to activity of several isoflavones (genistein, daidzein, prunetin, genistin, biochanin A) [38]. The presence of a phenol group at C5 was suggested to be the structural feature critical for the inhibitory activity of genistein on tyrosine kinases. However, two other phenol groups at C7 and C4' were also cited as important for interactions with kinases [38]. Importantly, the large substituent, such as the glucose occurring at C7 position in genistin, was indicated as a factor in eliminating the inhibitory activity of the molecule. However, here we showed that substitution of genistein with certain sugar moieties did not eliminate inhibitory activity of genistein, and all the tested compounds were capable of EGFR phosphorylation inhibition in the concentration dependent manner.

### 2.3. Inhibition of EGFR Phosphorylation in Cancer Cells Treated with Genistein Derivatives and Ionizing Radiation

**Figure 4:**
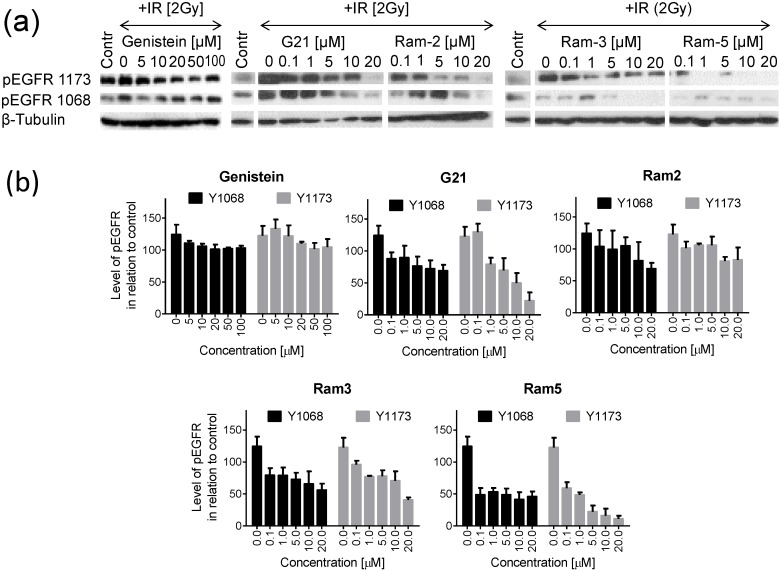
Phosphorylation of EGFR in HCT 116 cells treated with genistein or its derivatives for 24 h, irradiated with 2 Gy and recovered for 24 h. (**a**) Western blots showing pEGFR 1068, pEGFR 1173 level and β-tubulin (used as a loading control); (**b**) Charts presenting the results of densitometry. Bars show the mean values ± standard deviations of the band density normalized to the loading control in relation to untreated control (neither drug-treated nor irradiated) (Contr) normalized to the loading control. Data from at least three independent experiments.

It is well established that EGFR activity is stimulated by ionizing radiation. In order to determine whether genistein and its sugar derivatives are capable of suppressing the radiation induced phosphorylation of EGFR, we incubated the HCT 116 cells with genistein, G21, Ram-2, Ram-3 and Ram-5 for 24 h and then irradiated cells with 2 Gy, as described in “Material and Methods” section. The level of pEGFR was analyzed using SDS PAGE and immunoblotting. It can be seen that in cells not exposed to genistein or its derivatives, irradiation caused a significant increase of pEGFR (Y1068) and pEGFR (Y1173) (Figure 4). In cells preincubated with genistein derivatives for 24 h before irradiation, the level of pEGFR was lowered remarkably. In contrast, in cells pretreated with genistein and then irradiated, the reduction of EGFR phosphorylation was very weak. The compound Ram-5 was most effective in prevention of irradiation-induced EGFR phosphorylation.

Although the degree of clonogenicity loss caused by the treatment with different derivatives and EGFR phosphorylation inhibition were not matching perfectly, we observed a clear correlating tendency between their intensity. Inhibition of EGFR phosphorylation by glycosidic derivatives of genistein seems to play a very important role in cancer cell elimination after radiotherapy and makes them promising agents for sensitization of a tumor to this therapeutic modality. Diminished phosphorylation of several tyrosines of EGFR abrogates the survival strategy leading to transmission of the mitogenic signals and evasion of apoptosis that tumor cells normally adopt after irradiation [2]. In our western blotting analyses all the tested genistein derivatives inhibited phosphorylation of tyrosines Y1068 and Y1173 in the EGFR, which may have important implications for cell fate after radiotherapy. These tyrosines are essential for binding the SH2 domain of Grb2 and provide docking sites for Shc [39,40]. Inhibiting interactions with those adaptor proteins hampers initiation of the signaling pathway of MAP kinase [41,42], thus stopping mitogenic stimulation. The capacity of EGFR inhibition seems to be of much relevance to clonogenic death after irradiation. The tested genistein derivatives decreased the level of phosphorylated EGFR in irradiated cells more profoundly than genistein. This correlated with higher antiproliferative effects in cells treated by the combination: genistein derivatives/irradiation when compared to genistein/irradiation.

**Figure 5:**
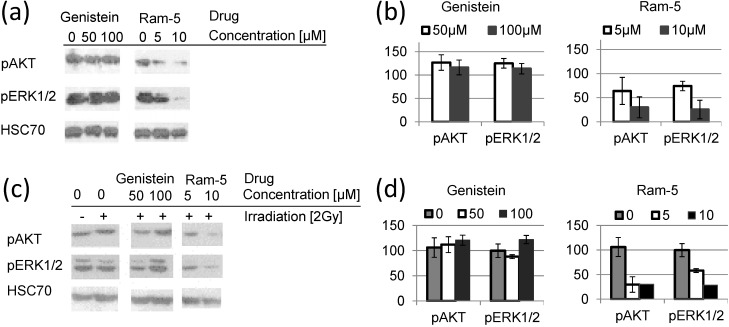
Phosphorylation of AKT and ERK1/2 in HCT 116 cells treated with genistein or its derivative Ram-5 for 24 h without (**a**,**b**) or with subsequent irradiation (**c**,**d**). (a) Western blots showing pAKT, pERK1/2 and HSC70 (used as a loading control); (b) charts presenting the results of densitometry. Bars show the mean values ± standard deviations of the band density normalized to the loading control in relation to untreated control normalized to the loading control; (c) Western blots showing pAKT, pERK1/2 and HSC70 (used as a loading control); (d) Charts presenting the results of densitometry. Bars show the mean values ± standard deviations of the band density normalized to the loading control in relation to untreated control (neither drug-treated nor irradiated), normalized to the loading control. Data from at least three independent experiments.

Next, we compared the activation of AKT and ERK in irradiated cells pretreated with genistein and the most potent derivative, Ram-5. The results presented in Figure 5. show that Ram-5 treatment, in contrast to genistein, leads to concurrent and prolonged inhibition of pAKT and pERK. The inhibitory effect observed after treatment of cells with Ram-5 also pertains after irradiation. Treatment of cells with genistein alone did not reduce the level of activated AKT and ERK1/2 (Figure 5). Moreover, we observed upregulation of activated AKT and ERK1/2 24 h after irradiation of the cells pretreated with genistein.

Synergistic inhibition of HCT 166 cell proliferation by a combination of genistein derivatives and ionizing radiation has profound implications for further studies. This cell line expresses a mutated form of KRAS [43], playing a crucial role in cell proliferation, survival and metastasis in colon cancer [44]. KRAS is an intermediate signal transduction element in the EGFR pathway, activating MAPK and ERK, thus inhibition of EGFR only will not cause effective shutdown of the downstream signaling. For that reason, KRAS mutation is regarded as a contraindication for therapeutic use of EGFR inhibitors [45,46]. Our discovery that the Ram-5 compound, in contrast to genistein, inhibited constitutively active ERK1/2 and AKT makes this compound a much more promising agent for sensitization of cells to radiotherapy than was the parent isoflavone. Ionizing radiation causes compensatory activation of AKT and ERK1/2, fueling the survival machinery and protecting cells from toxic effects of the treatment. Moreover, ERK1/2 activation may promote the release of paracrine regulators, such as hereguline, causing secondary EGFR activation in irradiated cells [43]. The observed concurrent inhibition of EGFR, AKT and ERK1/2 by Ram-5 abrogates prosurvival signaling on many levels, thus potentiating the effects of radiation much more effectively than genistein. Although many reports show that genistein inhibits activity of Akt and ERK1/2 we did not observe this effect in the HCT 116 cell line. Our results showing the lack of genistein effects on pERK and pAkt in HCT 116 cells having KRAS mutation, and constitutively activated ERK1/2 and Akt, contribute to the concept that ERK1/2 and Akt inhibition by genistein is most likely a secondary effect of EGFR inhibition and occurs in cells with wild type KRAS. In this cellular context, the ability of Ram-5 to decrease the level of both pERK and pAKT suggests interaction of the compound with signaling elements downstream of KRAS. Apparently, Ram-5 exhibits an additional mode of activity to that described for genistein, but the mechanistic role of that compound in ERK1/2 and AKT signaling requires further studies.

The use of small TKIs gains importance due to the fact, that clinically accepted antibodies targeting EGFR are not effective against tumors expressing the truncated form of EGFR, such as the mutated variant III of this receptor (EGFRvIII) [47,48]. Better control of cell proliferation may be achieved by treatment with combination of TKIs with different mode of action, *i.e.*, the EGFR targeting antibodies together with small inhibitors affecting intracellular domain [49,50,51]. The use of genistein in combination with ionizing radiation seems to be a promising option, although, on the other hand, there are some controversies about therapeutic use of genistein. Recent discoveries indicate that EGFR-dependent signaling pathways may be involved in stimulation of cancer cell proliferation by low concentrations of genistein [52,53,54]. The enhancement of cancer cell proliferation at low concentrations of this isoflavonoid is sometimes used as an argument against genistein supplementation [52]. The compound Ram-5 attracts attention as a drug potentially useful for radio-sensitizing colon cancer cells due to the better characteristics of EGFR inhibition when compared with genistein. This compound does not stimulate EGFR phosphorylation at low concentration, in contrast to genistein, so it may have a better safety profile *in vivo*. In this context, the use of new derivatives of genistein lacking those low-concentration stimulatory effects on EGFR should not be controversial. *In vitro* results from the work here will create the basis for animal study to evaluate the efficacy of genistein derivatives in conjunction with radiation therapy *in vivo*.

## 3. Experimental Section

### 3.1. Chemicals

Genistein (5,7-dihydroxy-3-(4-hydroxyphenyl)chromen-4-one) was obtained from Pharmaceutical Research Institute (Warsaw, Poland). Genistein derivatives: G21 (7-*O*-(2,3,4,6-tetra-*O*-acetyl-β-d-galactopyranosyl)-(1→4)-(6-*O*-acetyl-hex-2-ene-α-d-erythropyranosyl)-3-(4-hydroksyphenyl)-chromen-4-one), Ram-2 (5-hydroxy-7-[(4-*O*-acetyl-2,3,6-trideoxy-α-l-erythrohex-2-enopyranosyl)-2-*O*-ethyl]-3-(4-hydroxyphenyl)chromen-4-one), Ram-3 (5-hydroxy-7-[(4-*O*-acetyl-2,3,6-trideoxy-α-l-erythrohex-2-enopyranosyl)-3-*O*-propyl]-3-(4-hydroxyphenyl)chromen-4-one) and Ram-5 (5-hydroxy-7-[(4-*O*-acetyl-2,3,6-trideoxy-α-l-erythrohex-2-enopyranosyl)-5-*O*-pentyl]-3-(4-hydroxyphenyl)chromen-4-one) were synthesized as described previously [25,26]. The structures of the tested compounds are shown in Figure 1. Genistein, G21, Ram-2, Ram-3 and Ram-5 stock solutions were prepared in dimethyl sulfoxide (DMSO), stored at −20 °C and diluted before use. Final concentration of DMSO in culture media did not exceed 0.5%.

### 3.2. Cell Lines and Culture Conditions

The HCT 116 colon cancer cell line and DU 145 prostate cancer cell line obtained from ATCC (American Type Culture Collection, Rockville, MD, USA) were routinely cultured in an RPMI 1640 medium supplemented with 10% fetal bovine serum (FBS, ICN Pharmaceuticals, (Costa Mesa, CA, USA) and 1 μg/mL gentamicin (KRKA, Novo Mesto, Slovenia), at 37 °C in a humidified atmosphere containing 5% CO_2_ in the air. The cells were split every three days at 90% confluence. The cells were detached by rinsing with 0.02% ethylenediamine tetraacetic acid (EDTA) followed by incubation with 0.25% trypsin, and they were stored as frozen stocks in liquid nitrogen.

### 3.3. Clonogenic Assay

The cells were seeded at the density 1000 cells per a 6 cm plate (Nunc, Roskilde, Denmark). After 24 h, the medium was aspirated and replaced with a fresh one containing genistein or its derivatives. After 24 h, the treatment medium was replaced with a fresh one and cells were irradiated as described below.

### 3.4. Irradiation of Cells

The cells were irradiated with a 6 MV photon beam generated by a linear accelerator Clinac 2300 (Palo Alto, CA, USA) at a dose rate of 100 monitory units/min. The field was 30 cm × 30 cm, source-specimen distance (equivalent to source-skin distance, SSD) was 100.5 cm. The doses 1 Gy, 2 Gy or 4 Gy were equivalent to the doses measured under dosimetric conditions.

### 3.5. CI Calculation

Calculations of drug concentration/irradiation dose-effect and combination indices for different effects were calculated with Calcusyn software (Biosoft) using the median effect method described by Chou and Talalay [55]. The experiments enabling analysis of combination effects of drugs and irradiation had the checkerboard (latin square) design. In short, cells were treated with three serial growing concentrations of the drugs or the doses of radiation alone (the row and the column of the square formed an orthogonal series) or with the combination of the drug/radiation, used at the concentration/dose series forming the diagonal of the checkerboard.

### 3.6. Western Blotting

The cells were seeded in 10 cm culture plates and left for 24 h to allow the cells to attach to the bottom. They were then treated with a series of growing concentration of genistein, G21, Ram-2, Ram-3, Ram-5 for 24 h. In experiments testing the effect of combined treatment of genistein or its derivatives and ionizing radiation on EGFR phosphorylation medium containing isoflavones was aspirated and replaced with fresh medium without the tested drug, cells were irradiated with 2 Gy, as described above, and incubated for additional 24 h. Prior to the protein sample preparation cells were exposed to epithelial growth factor (EGF) at 50 ng mL in 0.1% bovine serum albumin (BSA) for 15 min and were lysed with the protein lysis buffer: Tris buffer pH 7.5 (50 mM), (1%) NP40 (Tergitol-type NP-40, nonyl phenoxylpolyethoxylethanol), NaCl (150 mM), ethylenediaminetetraacetic acid (EDTA, 1 mM), protease inhibitor solution (1:100, CompleteTM Roche, Basel, Switzerland) and phosphatase inhibitor solution (1:100; Sigma-Aldrich, Saint Louis, MO, USA). Subsequently, they were scraped, transferred to microtubes and centrifuged at 20,000 *g* for 10 min at 4 °C. The supernatant was collected, and the total protein content was quantified using a Bradford protein assay kit. The cell lysates were stored in −70 °C until use, and the proteins were separated by SDS/PAGE in 8% polyacrylamide gel at 100 V for 1.5 h, and transferred to nitrocellulose membrane (BA85; Schleicher and Schuell, Dassel, Germany). The blots were washed three times in TTBS (50 mM Tris/HCl, pH 7.5, 0.5% Tween-20, 0.15 M NaCl), blocked for 45 min at room temperature with 5% non-fat milk in TTBS and incubated overnight at 4 °C with the primary antibody. The antibodies used in the experiments were as follows: rabbit anti-EGFR (Cell Signaling, Danvers, MA, USA), rabbit and mouse anti-phospho-EGFR Y1068 (Cell Signaling), EGFR Y1173 (Millipore, Darmstadt, Germany), pAKT (Cell Signaling), pERK1/2 (Millipore), mouse-anti-β-tubulin (Sigma-Aldrich, Saint Louis, MO, USA), mouse-anti-actin (Cell Signaling), HSC70 (Santa Cruz, Dallas, TX, USA). After washing with TTBS, the blots were incubated with anti-rabbit or anti-mouse secondary antibody conjugated with horseradish peroxidase (HRP) for 1.5 h at room temperature. They were then washed in TTBS and the bands were visualized using SuperSignal West Pico Chemiluminescent Substrate (Thermo Scientific, Rockford, IL, USA).

## 4. Conclusions

Genistein glycoconjugates potentiate effects of radiotherapy in synergistic or additive manner. These compounds prevent phosphorylation of EGFR induced by ionizing radiation and are potentially useful drugs for radio-sensitizing colon cancer cells.
